# Disparities in Exclusive Human Milk Feeding and Unintended Formula Supplementation Among Infants Discharged Following Birth at an Academic Medical Center

**DOI:** 10.3390/nu18193294

**Published:** 2026-10-08

**Authors:** Georgette Richardson, Robert D. Roghair, Kelly E. Wood, Aunum Akhter, Jennifer R. Bermick, Emily A. Spellman, Lora B. Albert, Temitope M. Awelewa

**Affiliations:** 1 Division of Pediatric Psychology, Department of Pediatrics, Carver College of Medicine, University of Iowa, Iowa City, IA 52242, USA; 2 Division of Neonatology, Department of Pediatrics, Carver College of Medicine, University of Iowa, Iowa City, IA 52242, USA; 3 Division of Hospital Medicine, Department of Pediatrics, Carver College of Medicine, University of Iowa, Iowa City, IA 52242, USA; 4 Nursing, Health Care Stead Family Children’s Hospital, University of Iowa, Iowa City, IA 52242, USA; 5 Lactation Services, Health Care Stead Family Children’s Hospital, University of Iowa, Iowa City, IA 52242, USA; 6 Division of General Pediatrics and Adolescent Medicine, Department of Pediatrics, Carver College of Medicine, University of Iowa, Iowa City, IA 52242, USA

**Keywords:** age, breastfeeding, education, equity, insurance, neonates, obstetrics, social determinants

## Abstract

Background: The mitigation of existing disparities in human milk intake is a shared responsibility. Health systems have emphasized antenatal decision making to increase the proportion of infants that receive Exclusive Human Milk (EHM). However, our recent focus group reported that inadequate communication and other systemic barriers were contributing to unintended formula supplementation. Methods: To explore the influence of sociodemographic factors on EHM intake, we analyzed records for 10,214 newborns discharged from our medical center. Results: While race or ethnicity and prenatal EHM intent were the best predictors of EHM, the lowest rates were seen among infants born to Muslim women or women with non-English language preference (NELP). Even when analysis was restricted to those with a prenatal plan for EHM, unintended formula use exceeded 50% among those that identified as Black, Muslim, or having a NELP. In exploratory analysis, the same predictors were associated with the lowest rates of pasteurized donor human milk (PDHM) utilization. Conclusions: Looking deeper than race and ethnicity, Muslim women and women with NELP have the lowest rates of PDHM utilization and the highest rates of unintended formula use at our institution. These findings can inform culturally responsive interventions focused on education and effective communication.

## 1. Introduction

Exclusive human milk (EHM) feeding is associated with reduced rates of infection, a lower rate of childhood obesity, and other health benefits [1,2]. Based on documented benefits to both women and their infants, the American Academy of Pediatrics and the World Health Organization recommend EHM for approximately 6 months [3,4]. However, nearly 20% of breastfed infants in the United States receive formula supplementation within the first 48 h after birth, and the national rate of EHM through 6 months is only 26%, a percentage that lags far behind the Healthy People 2030 goal of 42% [3].

Beyond the drive for an overall improvement in human milk utilization, EHM rates in the United States remain negatively impacted by pervasive health inequities. While non-modifiable factors such as race and ethnicity are present, with lower rates of EHM seen among people of color [5], it is important to contextualize those associations and reflect on the antecedents that preceded the persistent disparities [6]. In addition, social factors, such as income, insurance status or language preference have intersectional effects that hamper EHM utilization in the impacted populations [7,8].

Participation in efforts to mitigate inequities and improve human milk feeding rates is a shared responsibility. Healthcare systems emphasize antenatal shared decision making and often focus on the health benefits of human milk in those prenatal discussions, including encouragement to consider pasteurized donor human milk (PDHM) during the birth hospitalization to augment other strategies used to increase the proportion of infants that receive EHM [9,10,11,12]. The Ten Steps to Successful Breastfeeding has been promulgated by the Baby-Friendly Hospital Initiative to further improve breastfeeding outcomes, especially when the steps are coupled with community support [4,13]. For example, supplementation of maternal human milk feedings is generally discouraged with the rate of formula supplementation used as a measure of the quality of perinatal care [14]. When supplementation is medically indicated, PDHM is recommended as the preferred alternative to cow milk-based formulas [3,15]. Unfortunately, there are disparities present not just regarding the use of PDHM, but also regarding the availability of PDHM across hospitals [16,17].

While the cumulative effects of structural and social determinants of health can, by themselves, overwhelm maternal intent for EHM [6], birthing hospitals still need to critically assess whether they are meeting their own responsibility by using their resources to mitigate the impacts of as many impediments as possible. Therefore, in addition to considering demographic and socioeconomic factors, quality improvement initiatives are designed to consider barriers to successful actualization of prenatal maternal intent that might arise during hospitalization, including illness, cesarean delivery, and a discordance in preferred language between the family and the healthcare team [12,18,19].

We recently summarized the results of a focus group that detailed factors which contributed to reduced human milk feeding among an immigrant population that predominately received care within our healthcare system [20]. In addition to sociodemographic factors, participants identified perinatal education and communication, specifically regarding the benefits of breastfeeding and use of PDHM, as strong contributors towards unintended formula supplementation. Women in the focus groups expressed concern about misinformation from health care professionals, insufficient education on benefits of breastfeeding and communication difficulties with the use of interpreters. Many participants felt maternal breast milk is personalized, were against the use of PDHM for their infants and would rather give their infants formula [20]. The lessons from that experience highlighted a need to better understand the patient populations that experience healthcare disparities within our catchment area. Recognizing a need to improve human milk utilization rates for our families throughout their time with us, we therefore established a multidisciplinary taskforce to identify barriers contributing to disparate within-hospital breastfeeding rates.

The primary objective of our study was to identify sociodemographic, cultural, and pregnancy-related factors associated with EHM feeding at hospital discharge among infants born at a large academic medical center. A secondary objective was to disaggregate maternal intent and outcomes by evaluating disparities in the actualization of EHM feeding among women with a documented prenatal intent for EHM. As part of ongoing quality improvement efforts, we aimed to use these results to inform culturally responsive strategies that promote equitable actualization of prenatal feeding intentions across patient populations.

## 2. Materials and Methods

Following IRB submission (IRB #202309332), the study was determined to be not human subjects research. We reviewed electronic medical records (EMRs) of all infants born at and discharged from the University of Iowa Health Care Stead Family Children’s Hospital between 1 October 2021 and 31 December 2024. Throughout that timeframe, there was an institutional expectation that all women received lactation support within 24 h of delivery, regardless of the location of their infant, and breast pumps were made available immediately after delivery with skin-to-skin contact strongly encouraged as soon as the infant’s status allowed. There are no restrictions on the use of PDHM for patients admitted to our hospitals, but a parent must provide verbal followed by written agreement that they have been informed about banked human milk and its use in feeding their infant before PDHM can be utilized, and the document to be signed (Parent Agreement to Feed Banked Human Milk from the Mother’s Milk Bank of Iowa) is only available to our families in English. The unit of analysis (N) was the individual infant with no correction for multiple births. Likewise, no infants were excluded based on maternal or infant illness or medication administration, and those variables were not tracked.

Any Human Milk (AHM; any intake of maternal milk or PDHM) or EHM (no infant formula other than that which could be added to human milk as a fortifier) was ascertained by EMRs documented intake of human milk versus formula. For infants discharged from units other than the neonatal intensive care unit (NICU), bottle feeding with mother’s own milk was not distinguished from bottle feeding with PDHM, and that precluded analysis of PDHM intake in those units. Sociodemographic variables that could be extracted from the EMRs and might be associated with EHM rates were identified by multidisciplinary taskforce recommendations. For continuous variables (maternal age and gestational age at delivery), the population’s median value was considered the reference cohort. For categorical variables (self-reported marital status, self-reported maternal education, self-reported race and ethnicity, primary payor, self-reported religion, self-reported preferred language, self-reported prenatal plan for newborn feeding, mode of delivery, number of infants delivered, and department that discharged the infant), the most common named category was considered the reference cohort. Regarding the categorization of race and ethnicity, individuals that self-reported “Hispanic” and any additional race or ethnicity other than “White” were included in the “more than one” category while those that self-reported only “Hispanic” or “Hispanic” and “White” were encoded as “Hispanic”. Potential associations of the 12 extracted sociodemographic variables with EHM or AHM were analyzed by univariate followed by multivariate binary logistic regression and then forward stepwise logistic regression for the entire cohort as well as the sub-cohort that had a prenatal plan for EHM. The associations of race and ethnicity with each of the other variables were assessed by univariate and bivariate logistic regression. In an exploratory analysis, due to the available data that restricted the sample to only the subset of infants discharged from the NICU, the association of the sociodemographic variables with PDHM was assessed by only univariate binomial logistic regression. All logistic regression was performed using IBM SPSS Statistics for Windows, version 31 (IBM Corp., Armonk, NY, USA). To further contextualize and visualize the inter-related associations between the sociodemographic variables, additional exploratory analysis was performed by Principal Component Analysis (PCA) with data-derived dichotomized variables using SigmaPlot version 14 (Systat Software, San Jose, CA, USA) with statistical significance defined by *p* < 0.05.

## 3. Results

Among N = 10,214 infants, the overall EHM and AHM rates at discharge were 60% and 87%, but significant disparities were present across the maternal variables of interest (Table 1). Regarding maternal age, the lowest odds of EHM and AHM were seen in dyads with a maternal age < 26 years, and by univariate but not multivariate analysis, maternal age > 34 years was associated with reduced odds of EHM. Married women had higher EHM and AHM rates than any other marital status. Any college education, especially advanced college beyond an initial degree, was strongly associated with increased EHM and AHM rates. While EHM rates were the highest for women that identified as White, AHM rates were highest among Asian women. Similarly, Muslim women had among the lowest rates of EHM and among the highest rates of AHM. Likewise, a non-English language preference (NELP) was associated with very low odds of EHM, but variable odds of AHM. Of note, among the Muslim population included in this study, 56% had a documented preference for English and 33% had a documented preference for Arabic, and their corresponding EHM rates were 33% and 12%, respectively.

While infants born at <39 weeks’ gestation had lower odds of receiving EHM and AHM, those born at >39 weeks had increased odds of receiving EHM and AHM. Families with a private payor had higher EHM and AHM rates than families with public payors such as Medicaid or Medicare. As expected, those with a prenatal intent for EHM had higher rates of EHM and AHM than those without that documented intent, and EHM or AHM rates were lower with C-section versus vaginal delivery and multiple infants (twins or triplets) versus singletons. Finally, infant discharge from any department other than the well nursery was associated with reduced odds of EHM but not AHM.

Forward stepwise regression was then performed to identify the strength of the associations of the overall variables with EHM utilizing probability for stepwise entry of 0.05 and removal of 0.10. All twelve variables under consideration helped to predict EHM (Table 2), led by prenatal intent, maternal race or ethnicity, primary payor, and language preference. The model correctly classified 75% of the total cases, an improvement from the baseline classification accuracy of 60%.

Among the 5325 women with a documented intent to provide EHM, the overall success rate was 72% with AHM utilization by 95%, but disparities were again noted across the maternal variables of interest (Table 3). High levels of statistical significance (*p* < 0.01 by multivariate analysis) and clinical significance (EHM rate < 50% despite a prenatal plan of EHM) were seen among women that identified as separated, Black, Muslim, or preferring a language other than English (Table 3).

By forward stepwise regression restricted to those with prenatal EHM intent, race or ethnicity, primary payor and preferred language were the best predictors of EHM success (Table 4). The stepwise algorithm reached its final model at Step 9 when the remaining variables (number of babies and maternal age) did not meet the entry criteria. The model correctly classified 77% of the total cases, an improvement from the baseline classification accuracy of 72%.

**Figure 1:**
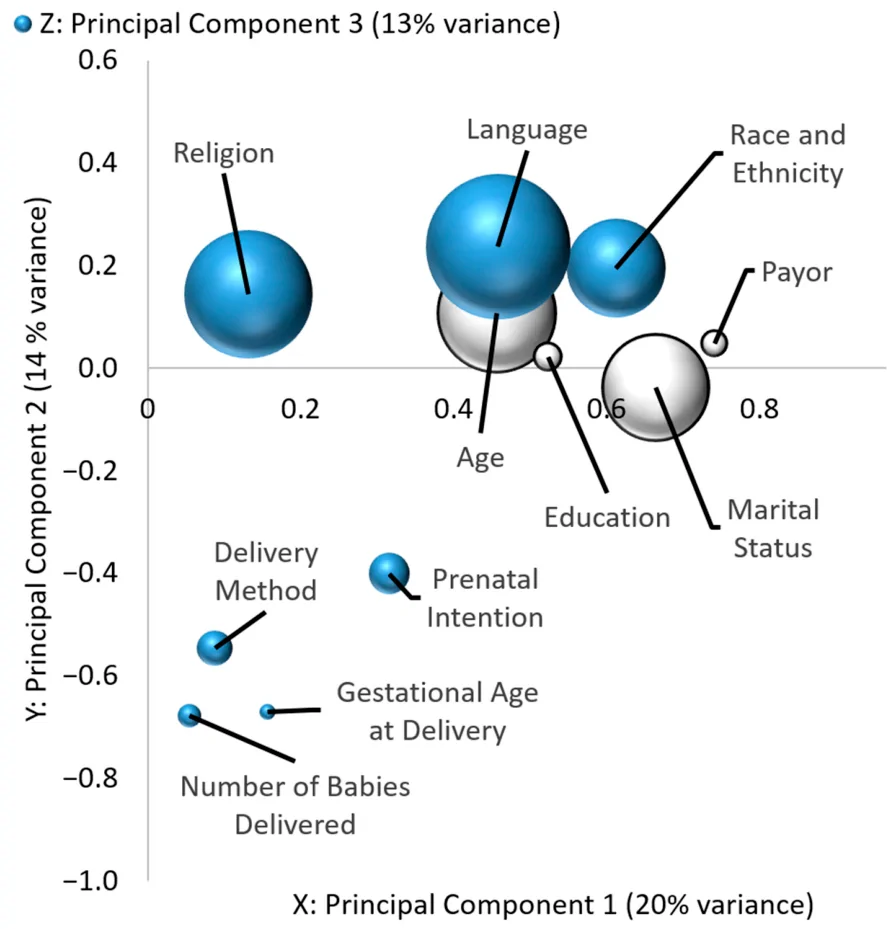
Principal component analysis of variables that can be used to predict Exclusive Human Milk (N = 10,214). The 3D bubble plot shows 1 of 3 components on each axis.

To visually depict the interrelationships between the sociodemographic variables associated with EHM, exploratory PCA was performed (Figure 1). To perform the analysis, the variables were first dichotomized based on the statistically significant results by univariate logistic regression, as shown in Table 1. The data-driven dichotomization was therefore: (1) maternal age 25 years or younger versus 26 years or older, (2) not self-identified as married versus married, (3) not self-identified as attending college versus any college, (4) not self-identified as person of color (POC) versus POC, (5) private versus not private primary payor, (6) not self-identified as Muslim versus Muslim, (7) self-identified NELP versus self-identified English language preference, (8) prenatal plan for EHM versus no self-identified plan for EHM, (9) cesarean section versus vaginal delivery, (10) gestational age at delivery of 38 weeks or younger versus 39 weeks or older, and (11) birth of a single infant versus multiple infants. The exploratory or hypothesis-generating PCA resulted in the 11 predictive variables being categorized into three Principal Components (PCs) that each had an Eigenvalue over 1 (Figure 1). PC-1 (*x*-axis) was dominated by baseline sociodemographic factors (payor, marital status, race/ethnicity, education level, and age), PC-2 (*y*-axis) was dominated by pregnancy-specific factors (number of infants delivered, gestational age at delivery, mode of delivery), and PC-3 (*z*-axis with direction and magnitude depicted by the color and size of the bubbles, respectively) added cultural factors (preferred language and religion). Prenatal intent appeared to be an important part of each component.

Among the variables within our population, maternal race or ethnicity was strongly associated with baseline sociodemographic variables (age, marital status, education, insurance status) and culture (language and religion) rather than pregnancy-specific variables such as mode of delivery or number of infants delivered (Figure 1 and Table 5).

Given the challenges that might occur with the PDHM consent for Muslim women and individuals with NELP, we performed an exploratory analysis to determine the rate of PDHM use within the NICU, the only department that separately documented maternal versus PDHM intake. For that subgroup of infants discharged from the NICU (N = 1605), 58% received PDHM, but significant variation was noted (Table 6), with the lowest rates of PDHM among Muslim women or those with NELP.

## 4. Discussion

This retrospective cohort study identified multiple sociodemographic, cultural, and pregnancy-related factors associated with EHM feeding at discharge, including insurance and marital status, and it reinforces the widespread assumption that a documented prenatal plan to use EHM remains a major predictor of postnatal EHM actualization. Unfortunately, this study also demonstrates that even when a shared decision is made to provide EHM, that common goal is often not achieved with inequities extending beyond maternal intention to encompass sociodemographic, cultural, and healthcare system factors. In our cohort, maternal race or ethnicity was the major predictor of unintended formula use; however, our findings indicate that race and ethnicity should not be viewed in isolation but rather considered in the context of social drivers of health.

Our findings reinforce the importance of socioeconomic factors in breastfeeding outcomes [21]. Younger maternal age, lower educational attainment, public insurance, and unmarried status were all associated with lower EHM rates. These characteristics likely reflect differences in access to breastfeeding education, social support, health literacy, and financial resources rather than differences in maternal intention [22,23]. A meta- analysis similarly showed low maternal literacy, maternal breastfeeding education, and infant separation as perinatal factors associated with breastfeeding in the immediate postpartum period [24]. Healthcare systems cannot directly modify these factors but can work towards mitigating their impact by providing individualized lactation support and access to community resources.

Pregnancy-specific factors, including cesarean delivery, preterm birth, multiple gestation, and NICU admission, were also associated with reduced EHM rates. These findings are also expected given the challenges associated with maternal-infant separation and medical instability. Nevertheless, families experiencing anticipated or unexpected complications may require intensified multidisciplinary support. Readily available breast pumps and lactation consultation protocols remain important evidence-based strategies to support successful human milk feeding in these populations [25,26].

Our study is relatively unique in demonstrating the major association between cultural factors, including language preference or religion, and the pattern of infant nutrition within the context of racial and ethnic disparities. Despite expressing a prenatal intention to exclusively provide human milk, 76% (250 of 328) of the women that preferred a language other than English experienced unintended formula supplementation. Language preference does not directly measure English proficiency or communication quality. However, language barriers may affect communication about EHM goals, PDHM consent, lactation support, and decisions regarding formula supplementation during hospitalization [18,19]. Even when interpreter services are available, inconsistent utilization and a lack of translated written material may reduce the quality of communication [27]. Health systems need to improve clinician training and promote a culture that is deliberate about consistently addressing language barriers by utilizing high-quality translation services [28,29].

Similarly, Muslim women experienced among the lowest rates of EHM and PDHM utilization. Religious beliefs surrounding milk kinship, PDHM acceptability, and family decision-making have been described in previous studies as important but surmountable considerations for Muslim families [30,31]. Our findings suggest that PDHM processes may not consistently provide culturally appropriate counseling regarding the perspectives of Muslim women, particularly when communication barriers coexist [32]. Collaborating with Muslim community leaders and culturally knowledgeable clinicians may improve informed decision-making while respecting individual beliefs and religious diversity.

An important strength of this study is the inclusion of more than 10,000 mother-infant dyads from a regional academic referral center, providing substantial statistical power to detect disparities across multiple demographic characteristics that were previously not apparent [33]. Furthermore, examining outcomes among women with documented prenatal EHM intent allowed distinguishing disparities in breastfeeding intention from disparities in achieving the intended feeding goal. This approach highlights healthcare system factors that may be amenable to quality improvement interventions rather than attributing disparities solely to maternal choice.

Several limitations should be considered when interpreting these findings. First, the retrospective design limits the ability to establish causal relationships. Second, PDHM use outside the NICU could not be distinguished from maternal milk because of documentation practices; however, previous studies have suggested disparities in PDHM utilization likely extend to the newborn nursery [9]. Third, the study was conducted at a single tertiary academic medical center, which may limit generalizability. Some subgroups were small, particularly Muslim women (*n* = 218 overall, 89 with a prenatal EHM plan, and 16 in the NICU). Compared with a national sample, a higher proportion of women in our cohort identified as White (70% vs. 52%), a similar proportion identified as Black (11% vs. 9%), and lower proportions identified as Hispanic (10% vs. 25%) or more than one race (3% vs. 8%) [5]. Despite these differences in composition, the sociodemographic disparities in EHM feeding observed in our study were generally consistent with national patterns of exclusive breastfeeding by race and ethnicity, education, marital status, and economic resources [5]. Finally, several potentially important variables, including maternal body mass index, maternal and infant illness, medication use, prior breastfeeding experience and health literacy, were not available for analysis. We also did not statistically account for possible clustering of multiple infants from the same pregnancy and instead treated each infant as a unique feeding outcome. It is also worth noting that a lack of documented prenatal feeding plan within the field we queried does not mean a feeding plan was not present in the free-text sections of the medical record or that no prenatal feeding plan was in place.

A central question raised by these findings is what occurs between the documentation of prenatal intent and its actualization at discharge. First, documented feeding intent for EHM was patterned by social determinants of health: women of color were less likely than White women to have a documented prenatal plan for EHM (44% vs. 56%) and more likely to have a documented plan for combined human milk and formula feeding (11% vs. 3.4%; aOR 2.34, 95% CI 1.88–2.92) (Table 5). Second, and most strikingly, among women who intended during pregnancy to provide EHM, fewer than half of women of color (48%), Muslim women (22%), and women with NELP (24%) ultimately achieved this goal. Together, these findings reveal inequities at both the point of intent and the point of follow-through, suggesting that interventions targeting only one stage may be insufficient.

These findings also reframe the intervention target. Universal breastfeeding promotion is not disparity-neutral. Our quantitative findings align closely with qualitative barriers previously described among immigrant families in our catchment area [20]. The disproportionately low rates of EHM feeding and PDHM utilization among women of color, Muslim women, and women with NELP are consistent with reported communication barriers, including gaps in interpreter access, limited lactation support, and cultural concerns regarding PDHM [20]. Together, the two studies characterize the same problem from complementary perspectives: the community needs assessment identified barriers experienced by families, whereas the present analysis quantifies their population-level impact. This convergence suggests that the observed disparities are unlikely to reflect maternal preference alone and instead point to potentially modifiable, communication-dependent gaps in perinatal support. It also highlights the potential value of culturally tailored, peer-led, and audiovisual educational resources delivered in families’ preferred languages—an approach participants themselves endorsed [20].

## 5. Conclusions

In conclusion, our findings suggest that disparities in EHM feeding arise not only from differences in maternal feeding intention, but also from the interaction of maternal intent with structural, cultural, and healthcare system factors. Children born to women of color, Muslim women, and women with NELP experienced disproportionately high rates of unintended formula supplementation, underscoring the need to move beyond universal breastfeeding promotion toward culturally responsive, equity-focused interventions. Such efforts should address communication barriers, improve access to PDHM, engage community leaders, and ensure that all families receive the support necessary to achieve their feeding goals [34]. Health systems that implement culturally responsive lactation support programs incorporating interpreter services, multilingual educational resources, and culturally tailored PDHM counseling may be well positioned to improve feeding outcomes. Future work should prospectively evaluate the effects of such programs on longer-term family and population health.

## Figures and Tables

**Table 1:** Sociodemographic variables associated with exclusive human milk (EHM) or any human milk (AHM) analyzed by univariate binary logistic regression with odds ratios (OR) or multivariate binary logistic regression with adjusted odds ratios (aOR) and corresponding 95% confidence intervals.

Sociodemographic Variable	N (%)	EHM	OR (95% CI)	aOR (95% CI)	AHM	OR (95% CI)	aOR (95% CI)
Overall	10,214 (100%)	60%			87%		
Maternal Age (years)							
<20	215 (2.1%)	43%	0.40 (0.29–0.55) **	0.84 (0.59–1.20)	73%	0.33 (0.23–0.48) **	0.61 (0.39–0.97) *
20 or 21	364 (3.6%)	44%	0.42 (0.33–0.54) **	0.70 (0.52–0.94) *	82%	0.56 (0.40–0.80) **	0.95 (0.63–1.44)
22 or 23	573 (5.6%)	45%	0.44 (0.35–0.55) **	0.69 (0.53–0.90) **	80%	0.49 (0.36–0.66) **	0.80 (0.56–1.15)
24 or 25	860 (8.4%)	55%	0.65 (0.53–0.80) **	0.84 (0.66–1.06)	85%	0.71 (0.53–0.96) *	0.96 (0.67–1.36)
26 or 27	1139 (11%)	62%	0.89 (0.74–1.08)	1.00 (0.80–1.24)	87%	0.84 (0.63–1.13)	0.87 (0.62–1.21)
28 or 29	1362 (13%)	63%	0.92 (0.76–1.10)	0.96 (0.77–1.19)	88%	0.93 (0.70–1.23)	0.94 (0.68–1.31)
30	762 (7.5%)	65%	Reference	Reference	89%	Reference	Reference
31 or 32	1498 (15%)	65%	0.99 (0.82–1.19)	1.04 (0.84–1.29)	88%	0.87 (0.66–1.14)	0.82 (0.59–1.13)
33 or 34	1238 (12%)	64%	0.97 (0.80–1.17)	0.95 (0.76–1.18)	88%	0.89 (0.67–1.19)	0.74 (0.53–1.03)
35 or 36	932 (9.1%)	60%	0.80 (0.65–0.97) *	0.88 (0.70–1.11)	87%	0.82 (0.61–1.10)	0.73 (0.51–1.03)
37 or 38	606 (5.9%)	58%	0.74 (0.60–0.93) **	0.87 (0.67–1.13)	89%	1.04 (0.73–1.46)	1.00 (0.67–1.48)
39 or 40	371 (3.6%)	53%	0.61 (0.48–0.79) **	1.00 (0.73–1.35)	85%	0.72 (0.50–1.04)	0.89 (0.57–1.37)
>40	294 (2.9%)	57%	0.71 (0.54–0.93) *	0.99 (0.71–1.36)	86%	0.73 (0.49–1.09)	0.87 (0.55–1.39)
Marital Status							
Married	6873 (67%)	67%	Reference	Reference	91%	Reference	Reference
Single	2558 (25%)	43%	0.38 (0.34–0.42) **	0.66 (0.58–0.75) **	76%	0.29 (0.26–0.33) **	0.48 (0.40–0.57) **
Life Partner	653 (6.4%)	49%	0.47 (0.40–0.55) **	0.76 (0.63–0.93) **	83%	0.48 (0.38–0.59) **	0.83 (0.63–1.09)
Separated	55 (0.5%)	42%	0.35 (0.21–0.61) **	0.65 (0.35–1.21)	75%	0.28 (0.15–0.51) **	0.51 (0.24–1.06)
Unknown	75 (0.7%)	47%	0.43 (0.27–0.68) **	1.09 (0.63–1.91)	76%	0.30 (0.18–0.52) **	0.48 (0.25–0.90) *
Maternal Education							
College	2236 (22%)	68%	Reference	Reference	90%	Reference	Reference
High School	1204 (12%)	40%	0.32 (0.27–0.36) **	0.61 (0.52–0.73) **	76%	0.34 (0.28–0.41) **	0.64 (0.51–0.82) **
Advanced College	1177 (12%)	75%	1.45 (1.24–1.70) **	1.31 (0.94–1.36)	93%	1.44 (1.11–1.88) **	1.09 (0.80–1.48)
Elementary School	38 (0.4%)	26%	0.17 (0.08–0.35) **	0.68 (0.28–1.62)	76%	0.35 (0.16–0.74) **	0.43 (0.18–1.03)
Middle School	28 (0.3%)	46%	0.41 (0.20–0.88) *	0.82 (0.33–2.05)	82%	0.50 (0.18–1.32)	0.80 (0.23–2.76)
Unknown	5531 (54%)	58%	0.65 (0.59–0.72) **	0.86 (0.76–0.97) *	86%	0.68 (0.58–0.80) **	0.83 (0.69–1.00) *
Race and Ethnicity							
White	7164 (70%)	69%	Reference	Reference	88%	Reference	Reference
Black	1149 (11%)	22%	0.13 (0.11–0.15) **	0.34 (0.28–0.41) **	77%	0.44 (0.38–0.52) **	0.82 (0.66–1.02)
Hispanic	974 (10%)	45%	0.37 (0.32–0.42) **	0.66 (0.55–0.79) **	86%	0.83 (0.68–1.00)	1.28 (0.97–1.69)
Asian	364 (3.6%)	48%	0.41 (0.33–0.51) **	0.37 (0.29–0.48) **	93%	1.80 (1.19–2.72) **	1.53 (0.93–2.50)
More than One	327 (3.2%)	55%	0.55 (0.44–0.68) **	0.85 (0.66–1.09)	83%	0.63 (0.47–0.84) **	0.90 (0.64–1.27)
Native Hawaiian/Pacific Islander	35 (0.3%)	17%	0.09 (0.04–0.23) **	0.24 (0.09–0.63) **	54%	0.16 (0.08–0.31) **	0.33 (0.15–0.70) **
American Indian/Alaska Native	32 (0.3%)	50%	0.45 (0.23–0.91) *	0.75 (0.34–1.62)	84%	0.72 (0.28–1.87)	1.32 (0.42–4.10)
Unknown	169 (1.7%)	54%	0.54 (0.40–0.73) **	0.85 (0.59–1.23)	88%	0.94 (0.59–1.49)	0.91 (0.53–1.54)
Primary Payor							
Private	6719 (66%)	70%	Reference	Reference	91%	Reference	Reference
Not Private	3495 (34%)	40%	0.29 (0.27–0.32) **	0.60 (0.53–0.67) **	78%	0.35 (0.31–0.39) **	0.63 (0.54–0.75) **
Religion							
Christian	3610 (35%)	63%	Reference	Reference	88%	Reference	Reference
No Preference	3900 (38%)	59%	0.84 (0.76–0.92) **	0.90 (0.80–1.00)	85%	0.77 (0.68–0.89) **	0.91 (0.78–1.07)
None	1796 (18%)	59%	0.86 (0.76–0.96) *	0.87 (0.76–1.00)	86%	0.80 (0.67–0.94) **	0.83 (0.68–1.01)
Muslim	218 (2.1%)	24%	0.19 (0.14–0.26) **	0.38 (0.26–0.56) **	93%	1.80 (1.06–3.07) *	2.03 (1.06–3.88) *
Hindu	48 (0.5%)	52%	0.64 (0.36–1.13)	1.18 (0.61–2.28)	90%	1.14 (0.45–2.91)	0.52 (0.18–1.50)
Other	119 (1.2%)	60%	0.87 (0.60–1.26)	1.05 (0.68–1.63)	88%	1.00 (0.57–1.76)	0.80 (0.43–1.48)
Unknown	523 (5.1%)	59%	0.83 (0.69–1.00)	0.91 (0.73–1.15)	85%	0.77 (0.59–1.00)	0.86 (0.63–1.18)
Language Preference							
English	9325 (91%)	64%	Reference	Reference	87%	Reference	Reference
Spanish	335 (3.3%)	29%	0.24 (0.19–0.30) **	0.41 (0.30–0.55) **	85%	0.84 (0.62–1.13)	0.97 (0.64–1.49)
French	223 (2.2%)	9%	0.06 (0.04–0.09) **	0.14 (0.08–0.23) **	77%	0.51 (0.37–0.70) **	0.61 (0.41–0.90) *
Arabic	105 (1.0%)	10%	0.07 (0.04–0.13) **	0.22 (0.11–0.44) **	93%	2.10 (0.97–4.54)	1.60 (0.66–3.86)
Swahili	68 (0.7%)	4%	0.03 (0.01–0.08) **	0.08 (0.02–0.25) **	93%	1.89 (0.76–4.72)	3.06 (1.13–8.32) *
Other	158 (1.5%)	17%	0.12 (0.08–0.18) **	0.20 (0.13–0.32) **	80%	0.59 (0.40–0.88) *	0.57 (0.35–0.93) *
Prenatal Intent							
Human Milk	5325 (52%)	72%	Reference	Reference	95%	Reference	Reference
Formula	508 (5.0%)	5%	0.02 (0.01–0.03) **	0.02 (0.02–0.03) **	22%	0.01 (0.01–0.02) **	0.02 (0.01–0.02) **
Both	576 (5.6%)	26%	0.14 (0.11–0.17) **	0.21 (0.17–0.27) **	80%	0.20 (0.16–0.25) **	0.26 (0.20–0.33) **
Unknown	3805 (37%)	55%	0.49 (0.45–0.53) **	0.59 (0.53–0.65) **	84%	0.26 (0.22–0.30) **	0.32 (0.27–0.37) **
Delivery Method							
Vaginal	6832 (67%)	63%	Reference	Reference	88%	Reference	Reference
C-Section	3382 (33%)	53%	0.66 (0.61–0.72) **	0.75 (0.68–0.83) **	83%	0.66 (0.59–0.74) **	0.83 (0.72–0.96) *
Gestational Age (weeks)							
<35	886 (8.7%)	54%	0.76 (0.65–0.88) **	1.35 (1.06–1.70) *	80%	0.57 (0.47–0.70) **	0.66 (0.48–0.91) *
35 or 36	788 (7.7%)	49%	0.63 (0.54–0.74) **	0.84 (0.70–1.02)	80%	0.57 (0.47–0.70) **	0.67 (0.52–0.86) **
37 or 38	3020 (30%)	56%	0.83 (0.75–0.91) **	0.91 (0.81–1.03)	85%	0.79 (0.69–0.91) **	0.89 (0.75–1.06)
39	3598 (35%)	61%	Reference	Reference	88%	Reference	Reference
>39	1922 (19%)	70%	1.55 (1.38–1.74) **	1.42 (1.23–1.63) **	93%	1.80 (1.47–2.19) **	1.45 (1.15–1.82) **
Number of Babies							
Single	9606 (94%)	60%	Reference	Reference	87%	Reference	Reference
Multiple	608 (6%)	47%	0.58 (0.49–0.69) **	0.66 (0.54–0.81) **	80%	0.59 (0.48–0.73) **	0.90 (0.70–1.17)
Department							
Well Nursery	8253 (81%)	61%	Reference	Reference	87%	Reference	Reference
NICU	1605 (16%)	54%	0.74 (0.66–0.82) **	0.79 (0.67–0.93) **	83%	0.71 (0.61–0.82) **	1.22 (0.95–1.56)
Other	356 (3.5%)	49%	0.62 (0.50–0.77) **	0.67 (0.52–0.86) **	86%	0.86 (0.64–1.17)	1.39 (0.96–2.02)

* *p* < 0.05 and ** *p* < 0.01.

**Table 2:** Sociodemographic variables associated with EHM by Forward Stepwise Binary Logistic Regression with Entry into the Model Based on Likelihood Ratio.

Step	Variable	Wald	Nagelkerke R Square	*p*
1	Prenatal Intention	833.9	0.166	<0.001
2	Race and Ethnicity	799.8	0.266	<0.001
3	Primary Payor	295.6	0.296	<0.001
4	Language Preference	149.1	0.315	<0.001
5	Marital Status	92.1	0.324	<0.001
6	Gestational Age	71.7	0.332	<0.001
7	Delivery Method	36.1	0.335	<0.001
8	Maternal Education	44.6	0.340	<0.001
9	Religion	27.6	0.342	<0.001
10	Number of Babies	14.4	0.344	<0.001
11	Department Baby	14.9	0.345	<0.001
12	Maternal Age	21.3	0.347	0.046

**Table 3:** Sociodemographic variables associated with exclusive human milk (EHM) or any human milk (AHM), with restriction to only those with a documented prenatal plan for EHM, analyzed by univariate binary logistic regression with odds ratios (OR) or multivariate binary logistic regression with adjusted odds ratios (aOR) and corresponding 95% confidence intervals.

Sociodemographic Variable	N (%)	EHM	OR (95% CI)	aOR (95% CI)	AHM	OR (95% CI)	aOR (95% CI)
Overall	5325 (100%)	72%			95%		
Maternal Age (years)							
<20	104 (2%)	58%	0.43 (0.28–0.67) **	0.86 (0.52–1.41)	88%	0.31 (0.14–0.68) **	0.70 (0.30–1.62)
20 or 21	182 (3%)	58%	0.43 (0.30–0.62) **	0.72 (0.48–1.08)	95%	0.70 (0.32–1.56)	1.56 (0.67–3.64)
22 or 23	260 (5%)	59%	0.45 (0.32–0.63) **	0.64 (0.44–0.93) *	90%	0.38 (0.20–0.72) **	0.65 (0.33–1.30)
24 or 25	467 (9%)	67%	0.63 (0.47–0.84) **	0.77 (0.56–1.07)	96%	0.87 (0.45–1.66)	1.23 (0.62–2.45)
26 or 27	625 (12%)	74%	0.88 (0.66–1.17)	0.96 (0.70–1.31)	95%	0.78 (0.43–1.43)	0.78 (0.43–1.43)
28 or 29	758 (14%)	75%	0.95 (0.72–1.25)	0.98 (0.72–1.32)	97%	1.25 (0.66–2.35)	1.28 (0.66–2.48)
30	434 (8%)	76%	Reference	Reference	96%	Reference	Reference
31 or 32	789 (15%)	76%	0.99 (0.76–1.31)	1.00 (0.74–1.36)	96%	0.88 (0.49–1.59)	0.86 (0.47–1.60)
33 or 34	665 (13%)	75%	0.94 (0.71–1.25)	0.93 (0.68–1.27)	96%	1.09 (0.58–2.05)	1.00 (0.52–1.94)
35 or 36	483 (9%)	71%	0.79 (0.59–1.06)	0.92 (0.66–1.29)	95%	0.78 (0.41–1.47)	0.93 (0.48–1.83)
37 or 38	292 (6%)	68%	0.66 (0.48–0.92) *	0.73 (0.51–1.06)	96%	0.88 (0.42–1.83)	0.89 (0.41–1.92)
39 or 40	151 (3%)	72%	0.79 (0.52–1.20)	1.25 (0.77–2.03)	96%	0.99 (0.38–2.55)	1.36 (0.49–3.76)
>40	115 (2%)	70%	0.72 (0.46–1.14)	0.99 (0.59–1.66)	94%	0.63 (0.25–1.56)	0.65 (0.25–1.68)
Marital Status							
Married	3860 (72%)	76%	Reference	Reference	97%	Reference	Reference
Single	1109 (21%)	59%	0.44 (0.38–0.50) **	0.69 (0.57–0.83) **	90%	0.26 (0.20–0.34) **	0.43 (0.30–0.60) **
Life Partner	304 (6%)	63%	0.53 (0.42–0.68) **	0.75 (0.56–0.98) *	96%	0.68 (0.38–1.22)	0.93 (0.50–1.73)
Separated	23 (0.4%)	43%	0.24 (0.10–0.55) **	0.31 (0.13–0.75) **	78%	0.11 (0.04–0.30) **	0.17 (0.06–0.50) **
Unknown	29 (0.5%)	55%	0.38 (0.18–0.80) *	0.68 (0.28–1.64)	97%	0.85 (0.12–6.32)	1.16 (0.14–9.38)
Maternal Education							
College	1314 (25%)	78%	Reference	Reference	97%	Reference	Reference
High School	567 (11%)	54%	0.34 (0.28–0.42) **	0.58 (0.45–0.74) **	89%	0.22 (0.15–0.34) **	0.38 (0.24–0.60) **
Advanced College	696 (13%)	84%	1.47 (1.15–1.86) **	1.18 (0.91–1.52)	99%	1.93 (0.95–3.92)	1.35 (0.66–2.79)
Elementary School	15 (0.3%)	33%	0.14 (0.05–0.42) **	0.55 (0.15–2.09)	73%	0.08 (0.02–0.26) **	0.15 (0.03–0.65) *
Middle School	12 (0.2%)	67%	0.57 (0.17–1.92)	0.74 (0.20–2.67)	100%	not calculated	not calculated
Unknown	2721 (51%)	70%	0.66 (0.56–0.77) **	0.86 (0.72–1.01)	95%	0.54 (0.37–0.78) **	0.63 (0.42–0.93) *
Race and Ethnicity							
White	3981 (75%)	79%	Reference	Reference	96%	Reference	Reference
Black	437 (8%)	32%	0.12 (0.10–0.15) **	0.30 (0.23–0.39) **	87%	0.25 (0.18–0.34) **	0.45 (0.30–0.68) **
Hispanic	461 (9%)	57%	0.35 (0.28–0.42) **	0.58 (0.46–0.75) **	93%	0.53 (0.35–0.79) **	0.77 (0.48–1.24)
Asian	189 (3.5%)	53%	0.30 (0.22–0.40) **	0.29 (0.20–0.41) **	96%	0.96 (0.44–2.07)	0.85 (0.34–2.14)
More than One	148 (2.8%)	66%	0.52 (0.36–0.73) **	0.79 (0.54–1.15)	97%	1.05 (0.42–2.60)	1.89 (0.74–4.80)
Native Hawaiian/Pacific Islander	8 (0.2%)	25%	0.09 (0.02–0.44) **	0.18 (0.03–0.97) *	63%	0.06 (0.01–0.26) **	0.21 (0.04–1.12)
American Indian/Alaska Native	13 (0.2%)	46%	0.51 (0.33–0.80) **	0.33 (0.10–1.05)	92%	0.44 (0.06–3.41)	1.01 (0.12–8.79)
Unknown	88 (2%)	66%	0.51 (0.33–0.80) **	0.90 (0.53–1.54)	94%	0.61 (0.24–1.53)	0.61 (0.23–1.61)
Primary Payor							
Private	3799 (71%)	78%	Reference	Reference	97%	Reference	Reference
Not Private	1526 (29%)	56%	0.37 (0.32–0.42) **	0.71 (0.61–0.84) **	91%	0.34 (0.26–0.44) **	0.73 (0.53–1.01)
Religion							
Christian	1894 (36%)	74%	Reference	Reference	96%	Reference	Reference
No Preference	2031 (38%)	71%	0.87 (0.75–1.00) *	0.92 (0.79–1.08)	95%	0.83 (0.62–1.13)	0.93 (0.68–1.28)
None	946 (18%)	72%	0.92 (0.77–1.09)	0.91 (0.75–1.10)	96%	0.95 (0.65–1.39)	0.97 (0.65–1.45)
Muslim	89 (2%)	22%	0.10 (0.06–0.17) **	0.17 (0.10–0.31) **	94%	0.74 (0.29–1.88)	0.98 (0.31–3.10)
Hindu	22 (0.4%)	64%	0.61 (0.26–1.47)	1.87 (0.68–5.11)	91%	0.44 (0.10–1.92)	0.40 (0.07–2.25)
Other	75 (1%)	65%	0.66 (0.40–1.07)	0.92 (0.53–1.61)	91%	0.43 (0.19–0.96) *	0.54 (0.22–1.32)
Unknown	268 (5%)	73%	0.95 (0.71–1.27)	1.02 (0.74–1.42)	96%	1.03 (0.54–1.96)	1.08 (0.54–2.15)
Language Preference							
English	4997 (94%)	75%	Reference	Reference	96%	Reference	Reference
Spanish	127 (2%)	41%	0.23 (0.16–0.34) **	0.41 (0.26–0.63) **	94%	0.79 (0.36–1.70)	1.28 (0.51–3.21)
French	72 (1%)	8%	0.03 (0.01–0.07) **	0.08 (0.03–0.18) **	85%	0.25 (0.13–0.49) **	0.64 (0.29–1.42)
Arabic	37 (0.7%)	14%	0.05 (0.02–0.14) **	0.28 (0.10–0.80) *	92%	0.52 (0.16–1.70)	0.80 (0.19–3.42)
Swahili	27 (0.5%)	4%	0.01 (0.00–0.10) **	0.04 (0.01–0.31) **	93%	0.57 (0.14–2.43)	1.90 (0.39–9.15)
Other	65 (1%)	22%	0.09 (0.05–0.17) **	0.19 (0.10–0.36) **	89%	0.38 (0.17–0.84) *	0.58 (0.23–1.48)
Delivery Method							
Vaginal	3841 (72%)	74%	Reference	Reference	96%	Reference	Reference
C-Section	1484 (28%)	65%	0.64 (0.57–0.73) **	0.67 (0.58–0.78) **	93%	0.51 (0.40–0.66) **	0.63 (0.48–0.84) **
Gestational Age (weeks)							
<35	204 (4%)	68%	0.85 (0.62–1.16)	1.32 (0.87–2.00)	92%	0.50 (0.29–0.87) *	0.51 (0.24–1.06)
35 or 36	322 (6%)	63%	0.67 (0.52–0.86) **	0.76 (0.57–1.01)	88%	0.34 (0.23–0.51) **	0.37 (0.23–0.58) **
37 or 38	1510 (28%)	68%	0.83 (0.72–0.96) *	0.85 (0.72–1.00) *	94%	0.78 (0.57–1.06)	0.79 (0.57–1.09)
39	1988 (37%)	72%	Reference	Reference	96%	Reference	Reference
>39	1301 (24%)	79%	1.52 (1.29–1.79) **	1.51 (1.25–1.81) **	98%	2.54 (1.60–4.05) **	2.22 (1.38–3.58) **
Number of Babies							
Single	5153 (97%)	72%	Reference	Reference	95%	Reference	Reference
Multiple	172 (3%)	67%	0.81 (0.59–1.13)	0.91 (0.62–1.34)	91%	0.50 (0.29–0.86) *	0.75 (0.40–1.40)
Department							
Well Nursery	4580 (86%)	73%	Reference	Reference	96%	Reference	Reference
NICU	576 (11%)	67%	0.75 (0.62–0.90) **	0.77 (0.61–0.98) *	93%	0.66 (0.46–0.94) *	1.20 (0.75–1.95)
Other	169 (3.2%)	59%	0.53 (0.39–0.72) **	0.55 (0.39–0.78) **	95%	0.93 (0.45–1.93)	1.83 (0.80–4.16)

* *p* < 0.05 and ** *p* < 0.01.

**Table 4:** Sociodemographic variables associated with exclusive human milk (EHM), with restriction to only those with a documented prenatal plan for EHM, by Forward Stepwise Binary Logistic Regression with Entry into the Model Based on Likelihood Ratio.

Step	Variable	Wald	Nagelkerke R Square	*p*
1	Race and Ethnicity	474.376	0.131	<0.001
2	Primary Payor	96.894	0.154	<0.001
3	Language Preference	83.391	0.181	<0.001
4	Gestational Age	58.554	0.195	<0.001
5	Marital Status	44.395	0.205	<0.001
6	Delivery Method	29.112	0.212	<0.001
7	Religion	35.901	0.221	<0.001
8	Maternal Education	31.749	0.228	<0.001
9	Department Baby	14.461	0.231	<0.001
	Number of Babies		0.16	0.16
	Maternal Age		0.62	0.62

**Table 5:** Sociodemographic variables associated with maternal race or ethnicity with all women that reported a race or ethnicity other than White categorized as a person of color (POC) and those with unknown race or ethnicity excluded. Data are analyzed by univariate binary logistic regression with odds ratios (OR) or multivariate binary logistic regression with adjusted odds ratios (aOR) and corresponding 95% confidence intervals.

Sociodemographic Variable	POC	White	% POC	OR (95% CI)	aOR (95% CI)
Overall	2881 (29%)	7164 (71%)	29%		
Maternal Age (years)					
<20	114 (4.0%)	100 (1.4%)	53%	4.01 (2.92–5.52) **	1.98 (1.37–2.87) **
20 or 21	147 (5.1%)	212 (3.0%)	41%	2.44 (1.86–3.20) **	1.50 (1.09–2.05) *
22 or 23	223 (7.7%)	339 (4.7%)	40%	2.31 (1.82–2.95) **	1.57 (1.18–2.09) **
24 or 25	266 (9.2%)	585 (8.2%)	31%	1.60 (1.28–2.00) **	1.27 (0.97–1.66)
26 or 27	312 (11%)	807 (11%)	28%	1.36 (1.10–1.69) *	1.22 (0.94–1.58)
28 or 29	338 (12%)	1005 (14%)	25%	1.18 (0.96–1.46)	1.20 (0.93–1.54)
30	166 (5.8%)	584 (8.2%)	22%	Reference	Reference
31 or 32	363 (13%)	1114 (16%)	25%	1.15 (0.93–1.41)	1.17 (0.91–1.51)
33 or 34	291 (10%)	924 (13%)	24%	1.11 (0.89–1.38)	1.16 (0.89–1.51)
35 or 36	276 (10%)	646 (9.0%)	30%	1.50 (1.20–1.88) **	1.42 (1.08–1.87) *
37 or 38	177 (6.1%)	415 (5.8%)	30%	1.50 (1.17–1.92) **	1.38 (1.01–1.88) *
39 or 40	125 (4.3%)	233 (3.3%)	35%	1.89 (1.43–2.49) **	1.41 (0.99–2.01)
>40	83 (2.9%)	200 (2.8%)	29%	1.46 (1.07–1.99) *	1.27 (0.87–1.86)
Marital Status					
Married	1522 (53%)	5231 (73%)	23%	Reference	Reference
Single	1073 (37%)	1453 (20%)	42%	2.54 (2.30–2.80) **	1.98 (1.72–2.27) **
Life Partner	233 (8.1%)	412 (5.8%)	36%	1.94 (1.64–2.31) **	1.31 (1.05–1.63) *
Separated	18 (0.6%)	36 (0.5%)	33%	1.72 (0.97–3.04)	0.99 (0.50–1.99)
Unknown	35 (1.2%)	32 (0.4%)	52%	3.76 (2.32–6.09) **	2.08 (1.11–3.92) *
Maternal Education					
College	432 (15%)	1770 (25%)	20%	Reference	Reference
High School	515 (18%)	679 (9.5%)	43%	3.11 (2.66–3.63) **	1.44 (1.19–1.73) **
Advanced College	183 (6.4%)	978 (14%)	16%	0.77 (0.63–0.93) **	1.06 (0.85–1.31)
Elementary School	28 (1.0%)	10 (0.1%)	74%	11.47 (5.53–24) **	1.32 (0.35–5.01)
Middle School	12 (0.4%)	14 (0.2%)	46%	3.51 (1.61–7.65) **	1.61 (0.58–4.44)
Unknown	1711 (59%)	3713 (52%)	32%	1.89 (1.68–2.13) **	1.24 (1.08–1.43) **
Primary Payor					
Private	1238 (43%)	5390 (75%)	19%	Reference	Reference
Not Private	1643 (57%)	1774 (25%)	48%	4.03 (3.68–4.42) **	2.33 (2.05–2.64) **
Religion					
Christian	990 (34%)	2596 (36%)	28%	Reference	Reference
No Preference	1032 (36%)	2816 (39%)	27%	0.96 (0.87–1.06)	0.91 (0.81–1.03)
None	472 (16%)	1286 (18%)	27%	0.96 (0.85–1.09)	0.95 (0.82–1.11)
Muslim	158 (5.5%)	39 (0.5%)	80%	10.6 (7.4–15.2) **	8.75 (5.81–13.16) **
Hindu	45 (1.6%)	0 (0.00%)	100%	not determined	not determined
Other	44 (1.5%)	75 (1.0%)	37%	1.54 (1.05–2.25) *	1.33 (0.85–2.10)
Unknown	140 (4.9%)	352 (4.9%)	28%	1.04 (0.85–1.29)	0.83 (0.64–1.08)
Language Preference					
English	2058 (71%)	7144 (100%)	22%	Reference	Reference
Spanish	328 (11%)	1 (0.01%)	100%	1139 (160–8112) **	833 (117–5954) **
French	213 (7.4%)	0 (0.00%)	100%	not determined	not determined
Arabic	83 (2.9%)	8 (0.11%)	91%	36 (17–75) **	8.71 (3.96–19.18) **
Swahili	66 (2.3%)	0 (0.00%)	100%	not determined	not determined
Other	133 (4.6%)	11 (0.15%)	92%	42 (23–78) **	42 (22–79) **
Prenatal Intent					
Human Milk	1256 (44%)	3981 (56%)	24%	Reference	Reference
Formula	163 (5.7%)	342 (4.8%)	32%	1.51 (1.24–1.84) **	1.11 (0.88–1.39)
Both	323 (11%)	246 (3.4%)	57%	4.16 (3.49–4.97) **	2.34 (1.88–2.92) **
Unknown	1139 (40%)	2595 (36%)	31%	1.39 (1.27–1.53) **	1.09 (0.97–1.22)
Delivery Method					
Vaginal	1909 (66%)	4808 (67%)	28%	Reference	Reference
C-Section	972 (34%)	2356 (33%)	29%	1.04 (0.95–1.14)	1.04 (0.93–1.17)
Gestational Age (weeks)					
<35	259 (9.0%)	615 (8.6%)	30%	1.06 (0.90–1.25)	1.69 (1.28–2.23) **
35 or 36	225 (7.8%)	555 (7.7%)	29%	1.06 (0.86–1.21)	1.06 (0.85–1.32)
37 or 38	888 (31%)	2081 (29%)	30%	1.07 (0.96–1.19)	1.10 (0.96–1.25)
39	1007 (35%)	2531 (35%)	28%	Reference	Reference
>39	502 (17%)	1382 (19%)	27%	0.91 (0.81–1.04)	0.97 (0.83–1.13)
Number of Babies					
Single	2729 (95%)	6713 (94%)	29%	Reference	Reference
Multiple	152 (5.3%)	451 (6.3%)	25%	0.83 (0.69–1.00)	0.83 (0.65–1.05)
Department					
Well Nursery	2362 (82%)	5747 (80%)	29%	Reference	Reference
NICU	418 (15%)	1170 (16%)	26%	0.87 (0.77–0.98) *	0.65 (0.53–0.80) **
Other	101 (3.5%)	247 (3.4%)	29%	1.00 (0.79–1.26)	0.75 (0.56–1.01)

* *p* < 0.05 and ** *p* < 0.01.

**Table 6:** Sociodemographic variables associated with the use of pasteurized donor human milk (PDHM) during admission to the NICU analyzed by univariate binary logistic regression with odds ratios (OR) and corresponding 95% confidence intervals.

Sociodemographic Variable	N (%)	PDHM	OR (95% CI)
Overall	1605 (100%)	58%	
Maternal Age (years)			
<20	38 (2%)	47%	0.71 (0.34–1.50)
20 or 21	64 (4%)	66%	1.51 (0.79–2.88)
22 or 23	81 (5%)	64%	1.42 (0.78–2.58)
24 or 25	160 (10%)	61%	1.25 (0.75–2.07)
26 or 27	164 (10%)	52%	0.87 (0.53–1.43)
28 or 29	197 (12%)	56%	1.02 (0.63–1.65)
30	102 (6%)	56%	Reference
31 or 32	235 (15%)	54%	0.94 (0.59–1.51)
33 or 34	196 (12%)	64%	1.42 (0.87–2.32)
35 or 36	132 (8%)	57%	1.04 (0.62–1.75)
37 or 38	108 (7%)	65%	1.45 (0.83–2.54)
39 or 40	69 (4%)	59%	1.16 (0.62–2.15)
>40	59 (4%)	54%	0.94 (0.49–1.78)
Marital Status			
Married	979 (61%)	56%	Reference
Single	475 (30%)	60%	1.19 (0.95–1.48)
Life Partner	131 (8%)	72%	2.01 (1.34–3.00) **
Separated	10 (1%)	60%	1.19 (0.33–4.22)
Unknown	10 (1%)	40%	0.53 (0.15–1.88)
Maternal Education			
College	324 (20%)	58%	Reference
High School	181 (11%)	61%	1.15 (0.79–1.66)
Advanced College	122 (8%)	62%	1.20 (0.78–1.83)
Elementary School	4 (0.2%)	25%	0.24 (0.03–2.34)
Middle School	6 (0.4%)	17%	0.15 (0.02–1.25)
Unknown	968 (60%)	58%	0.99 (0.77–1.28)
Race and Ethnicity			
White	1170 (73%)	61%	Reference
Black	176 (11%)	48%	0.60 (0.43–0.82) **
Hispanic	122 (8%)	55%	0.80 (0.55–1.16)
Asian	49 (3%)	53%	0.74 (0.42–1.31)
More than One	58 (4%)	62%	1.07 (0.62–1.84)
Native Hawaiian/Pacific Islander	11 (1%)	55%	0.78 (0.24–2.58)
Unknown	17 (1%)	53%	0.73 (0.28–1.92)
Primary Payor			
Private	1008 (63%)	59%	Reference
Not Private	597 (37%)	58%	0.97 (0.79–1.19)
Religion			
Christian	613 (38%)	58%	Reference
No Preference	593 (37%)	60%	1.06 (0.84–1.34)
None	265 (17%)	58%	0.99 (0.74–1.32)
Muslim	16 (1%)	31%	0.33 (0.11–0.96) *
Hindu	10 (1%)	60%	1.08 (0.30–3.88)
Other	18 (1%)	83%	3.61 (1.03–12.6) *
Unknown	90 (6%)	53%	0.83 (0.53–1.29)
Language Preference			
English	1497 (93%)	60%	Reference
Spanish	38 (2%)	53%	0.75 (0.40–1.44)
French	34 (2%)	47%	0.60 (0.31–1.19)
Arabic	5 (0.3%)	0%	not determined
Swahili	6 (0.4%)	0%	not determined
Other	25 (2%)	32%	0.32 (0.14–0.74) **
Prenatal Intent			
Human Milk	576 (36%)	65%	Reference
Formula	75 (5%)	43%	0.41 (0.25–0.67) **
Both	70 (4%)	53%	0.62 (0.37–1.01)
Unknown	884 (55%)	56%	0.70 (0.56–0.87) **
Delivery Method			
Vaginal	662 (41%)	62%	Reference
C-Section	943 (59%)	56%	0.79 (0.65–0.97) *
Gestational Age (weeks)			
<35	802 (50%)	54%	0.70 (0.50–0.98) *
35 or 36	237 (15%)	64%	1.05 (0.70–1.58)
37 or 38	297 (19%)	61%	0.93 (0.63–1.38)
39	171 (11%)	63%	Reference
>39	98 (6%)	66%	1.18 (0.70–1.98)
Number of Babies			
Single	1300 (81%)	59%	Reference
Multiple	305 (19%)	57%	0.95 (0.74–1.23)

* *p* < 0.05 and ** *p* < 0.01.

## Data Availability

The data presented in this article are not readily available due to privacy restrictions for any use beyond the institution-specific purpose for which they were obtained.

## Abbreviations

The following abbreviations are used in this manuscript:

AHM	any human milk
EHM	exclusive human milk
EMRs	electronic medical records
NELP	non-English language preference
NICU	neonatal intensive care unit
PC	principal component
PCA	principal component analysis
PDHM	pasteurized donor human milk
POC	person of color
